# Neutrophil Gelatinase Associated Lipocalin Is an Early and Accurate Biomarker of Graft Function and Tissue Regeneration in Kidney Transplantation from Extended Criteria Donors

**DOI:** 10.1371/journal.pone.0129279

**Published:** 2015-06-30

**Authors:** Vincenzo Cantaluppi, Sergio Dellepiane, Michela Tamagnone, Davide Medica, Federico Figliolini, Maria Messina, Ana Maria Manzione, Massimo Gai, Giuliana Tognarelli, Andrea Ranghino, Caterina Dolla, Silvia Ferrario, Ciro Tetta, Giuseppe Paolo Segoloni, Giovanni Camussi, Luigi Biancone

**Affiliations:** 1 “A. Vercellone” Kidney Transplantation Center, Department of Medical Sciences, University of Torino, Azienda Ospedaliera “Città della Salute e della Scienza- Molinette”, Torino, Italy; 2 EMEALA Medical Board, Fresenius Medical Care, Bad Homburg, Germany; The University of Manchester, UNITED KINGDOM

## Abstract

**Background:**

Delayed graft function (DGF) is an early complication of kidney transplantation (KT) associated with increased risk of early loss of graft function. DGF increases using kidneys from extended criteria donors (ECD). NGAL is a 25KDa protein proposed as biomarker of acute kidney injury. The aim of this study was to investigate the role of NGAL as an early and accurate indicator of DGF and Tacrolimus (Tac) toxicity and as a mediator of tissue regeneration in KT from ECD.

**Methods:**

We evaluated plasma levels of NGAL in 50 KT patients from ECD in the first 4 days after surgery or after Tac introduction.

**Results:**

Plasma levels of NGAL at day 1 were significantly higher in DGF group. In the non DGF group, NGAL discriminated between slow or immediate graft function and decreased more rapidly than serum creatinine. NGAL increased after Tac introduction, suggesting a role as marker of drug toxicity. *In vitro*, hypoxia and Tac induced NGAL release from tubular epithelial cells (TEC) favoring an autocrine loop that sustains proliferation and inhibits apoptosis (decrease of caspases and Bax/Bcl-2 ratio).

**Conclusions:**

NGAL is an early and accurate biomarker of graft function in KT from ECD favoring TEC regeneration after ischemic and nephrotoxic injury.

## Introduction

Kidney Transplantation (KT) represents the most effective treatment for End Stage Renal Disease [1]. When compared to dialysis, KT improves patients survival, quality of life, cardiovascular morbidity and diminishes infectious complications [2,3]. However, several immunological and non-immunological causes lead to the deterioration of kidney graft function. Ischemia-reperfusion injury is the main cause of delayed graft function (DGF), an early complication of KT usually defined as the need for dialysis in the first week after the procedure. DGF is associated with the loss of graft function also due to an increased risk of acute rejection and it is a cause of reduced long term graft survival [4]. The incidence of DGF has been increasing over the last years for the use of kidneys from extended criteria donors (ECD) [5,6].

Recently, a broad variety of biomarkers including KIM-1, IL-18 and Neutrophil Gelatinase Associated Lipocalin (NGAL) have been proposed for the early detection of DGF. Among these, NGAL is a 25 KDa protein belonging to lipocalins, a family of proteins characterized by a hydrophobic cup-shaped binding site. NGAL has a high affinity for siderophora and it is involved in neutrophilic response to infections and in the processes of tissue differentiation and repair through iron chelation or delivering [7]. It has been previously shown that NGAL is expressed in tubular epithelial cells and that it is massively and rapidly up-regulated after ischemic, toxic or septic acute kidney injury (AKI) [8,9]. Moreover it has been recently demonstrated that NGAL can act as a tubular growth factor decreasing cell damage in experimental kidney ischemia-reperfusion injury [7,10].

The aim of this study is to evaluate whether plasma NGAL is an early and accurate biomarker of graft function in KT from ECD. For this purpose, we correlated plasma NGAL levels after KT with the incidence of DGF. In addition, plasma NGAL was measured after tacrolimus (Tac) administration to assess the early renal response to this nephrotoxic drug. *In vitro*, we investigated the potential regenerative effect of NGAL on cultured human tubular epithelial cells (TEC) exposed to hypoxia or incubated with therapeutic doses of Tac.

## Material and Methods

### Patients and clinical parameters

In this prospective monocentric study, we enrolled all consenting subjects who underwent KT from ECD from November 2013 until recruitment of 50 patients (September 2014) in the “A. Vercellone” Kidney Transplant Center in Torino, Italy. To assess if this selected group was representative of our population of patients receiving a KT from ECD, this group was compared to all the other patients fulfilling the same criteria in 2013–2014 (total 93). ECD were defined according to Cristal City criteria: donors over 60 years of age or over 50 with at least two of the following criteria: hypertension, serum creatinine > 132 mcmol/l (1.5mg/dl) or cerebro-vascular accident as cause of death [11]. Organ allocation was performed on the basis of histological criteria according to Karpinski et al. [12]. Induction immunosuppressive regimen was as it follows: basiliximab 20 mg on day 0 and day 4, mycophenolate mophetil 2g/die and steroids. Tac was given when serum creatinine reached 2.5 mg/dl (Tac target levels 12–15 ng/ml). The local ethic committee (Comitato Etico Interaziendale A.O.U. Città della Salute e della Scienza di Torino—A.O. Ordine Mauriziano—A.S.L. TO1) approved study protocol; a written consent was obtained from all KT recipients. None of the transplant donors were from a vulnerable population and all donors or next of kin provided written informed consent that was freely given in accordance to guidelines and standard procedures of AIRT (Associazione Inter-Regionale Trapianto) and Italian National Transplant Center (Centro Nazionale Trapianti—CNT).

Delayed Graft Function (DGF) was defined as the need for dialysis during the first week after KT [13]; non-DGF group was further divided in Slow Graft Function (SGF: serum creatinine > 3 mg/dl on day 6 after KT) and Immediate Graft Function (IGF: serum creatinine < 3 mg/dl on day 6 after KT) [14]. Ten patients with KT from living donors were enrolled as controls.

We evaluated the following clinical parameters: A) recipient variables: age, sex, BMI, hypertension, diabetes, dialysis duration (months), type of dialysis, peak PRA %, previous KT, type of nephropathy; B) donor variables: age, BMI, serum creatinine, eGFR, hypertension, diabetes, cause of death; C) KT procedure variables: cold ischemia time (CIT), HLA mismatches, pre-transplant Karpinski biopsy score. We also estimated DGF risk using the prediction nomogram proposed by Irish et al. [13] (http://www.transplantcalculator.com/DGF).

Moreover, a 10-year retrospective analysis (2002–2012) was performed to investigate the difference of DGF incidence between KT from ECD and non-ECD in our center.

### NGAL and Creatinine plasma levels

Patients plasma was collected immediately before surgery and in the first 24h after KT; samples were centrifuged (1000g for 10 minutes) and quickly frozen at −80°C. NGAL was measured by using a commercially available fluorimetric immunoassay (Triage kit, Alere Inc., San Diego, CA). All samples were analyzed in duplicate; intra-assay and inter-assay coefficient of variation were 11% and 13.5% respectively. NGAL was also evaluated in 4 consecutive days after KT (or until the start of dialysis in the DGF group), before and 24h after CNI introduction.

Serum creatinine was measured by Alkaline Picrate colorimetric assay, (Jaffè method); intra-assay and inter-assay coefficient of variation were 1.5% and 2.5% respectively.

### *In vitro* studies

#### Human Tubular Cell Isolation and Culture

Primary cultures of human tubular epithelial cells (TEC) were isolated and characterized as previously described [15]. TEC were grown in RPMI 1640 (Life Technologies, Grand Island, NY) containing 10% FCS (Hyclone, Logan, UT) and 2 mM glutamine (Life Technologies). In selected experiments TEC were cultured in hypoxic conditions for 24h into an airtight humidified chamber flushed with a gas mixture containing 5% CO_2_, 94% N_2_, and 2% O_2_ at 20 atm, 37°C [16] or with 20 ng/ml Tac (Sigma Aldrich, St. Louis, MO) for 24h. To investigate the activity of NGAL on TEC, 500ng/ml of recombinant NGAL (R&D Systems, Minneapolis, MN) were added to cell cultures. Appropriate recombinant NGAL concentration was chosen accordingly to values detected in our patients (see Results section) and after performing a dose-response TEC proliferation assay (S1 Fig).

#### Gene and protein expression in TECs cultured in different experimental conditions

Total RNA was isolated from TEC using the mirVana RNA isolation kit (Life Technologies). RNA was quantified by spectrophotometer (Nanodrop ND-1000, Nanodrop, Wilmington, DE, USA). First strand DNA was produced from 1μg of RNA using High cDNA Reverse Transcription Kit (Applied Biosystems, Foster City, CA, USA) [17]. Quantitative Real time PCR (qRT—PCR) was performed using the Power SYBR Green PCR Master Mix on a 96-well StepOnePlus Real Time System (Applied Biosystems). The following sequence-specific oligonucleotide primers were used (MWG-Biotech AG, Ebersberg, Germany):

*NGAL*: forward 5'-CCT CCG TCC TGT TTA GGA AAA A-3'; reverse 5'-TCG TTA ATC CAG GGT AAC TCT TAA TG-3';

*GAPDH (control housekeeping gene)*: forward, 5′-TGG AAG GAC TCA TGA CCA CAG T-3′; reverse 5′-CAT CAC GCC ACA GTT TCC C-3′;

Beta-Actin: forward, 5’-GAG TCC GGC CCC TCC AT-3’; reverse; 5’-GCA ACT AAG TCA TAG TCC GCC TAG A-3’.

In all experiments, 2.5μg of cDNA template were used and thermal-cycling conditions were as follows: activation of AmpliTaq Gold DNA Polymerase LD at 95°C for 10min, followed by 40 cycles of amplification at 95°C for 15sec and 60°C for 1min. Expression of mRNA levels was detected by using relative Ct quantification analysis as follows: **Delta**(Ct) = Ct target NGAL—Ct control housekeeping [18]. Relative NGAL expression was estimated by the formula: gene expression = 2 ^-Delta(Cτ)^. Both Beta-Actin and GAPDH were used as control housekeeping genes.

NGAL, megalin and ZO-1 protein expression on TEC was evaluated by FACS and immunofluorescence studies by using a rabbit polyclonal anti-human antibody (Santa Cruz Biotechnology) and an appropriate FITC-conjugated secondary antibody.

NGAL levels were also determined by ELISA in TEC supernatants in different experimental conditions (R&D Systems, Minneapolis, MN). Results were calculated after the generation of a standard curve with appropriate controls and given as averages ± 1 SD.

#### TEC cytotoxicity and apoptosis assays

TEC were cultured onto 24-well plates (Falcon Labware, Oxnard, CA) at a concentration of 5x10^4^ cells/well and incubated with different stimuli and 250 μg/ml XTT (Sigma Aldrich) in a medium without phenol red. The absorption values were measured in an automated spectrophotometer at 450 nm wavelength at different time points [19].

TEC cultured in different experimental conditions were subjected to TUNEL assay (ApopTag; Oncor, Gaithersburg, MD) in accordance with manufacturer’s instructions [19].

In selected experiments, TEC were seeded on 24-well plates and engineered to knock-down megalin, the NGAL receptor, by transfection with 80 pM of specific siRNA (S1 File). Transfection with 80 pM of irrelevant siRNA was used as experimental control according to the manufacturer’s instructions (Santa Cruz Biotechnology). After 48 h, the knock-down of megalin was verified by qRT-PCR, western blot (see above) and FACS analysis (S1 File) and cells were used for XTT and TUNEL assays as previously described.

#### Western blot analysis

For Western blot, cells were detached with EDTA and lysed with a 50-mM Tris-HCl buffer containing 1% Triton X-100, 10μM/ml leupeptin, 10μM PMSF, and 100 U/ml aprotinin. After centrifugation of TEC lysates at 15000g, protein contents of the supernatants were measured by the Bradford method: 30 μg of protein per lane was subjected to SDS-PAGE (10%) and electroblotted onto nitrocellulose membrane filters. Blots were subsequently blocked with 5% nonfat milk in 20 mM Tris-HCl (pH 7.5), 500 mM NaCl, and 0.1% Tween. Membranes were incubated overnight at 4°C with anti-bodies directed to human megalin, Bax and Bcl-2 (Santa Cruz Biotechnology) at a concentration of 500 ng/ml. After washing with 0.1% Tween, blots were stained for 1 h, RT with horse-radish peroxidase conjugated protein A (200 ng/ml; Amersham, Buckingamshire, UK), washed again with 0.1% Tween, developed with ECL detection reagents (Amersham), and exposed to X-Omat film (Eastman Kodak, Rochester, NY).

#### Caspase-3, -8, and -9 Activities

The activities of caspase-3, -8, and-9 were assessed by ELISA (Chemicon, Temecula, CA) on the basis of spectrophotometric detection of the cromophore p-nitroanilide (pNA) after cleavage from the labeled substrate DEVD-pNA, which is recognized by caspases. TEC lysates were diluted with appropriate reaction buffers, and DEVDpNA was added at final concentration of 50 M. Samples were analyzed with automated ELISA reader at a wavelength of 405 nm. Each experiment was performed in triplicate.

#### TEC functional assays

Trans-epithelial electrical resistance (TEER) was used as indicator of cell polarity. Cells were plated in transwells on collagen-coated polycarbonate membranes (Corning Costar Corp., Cambridge, MA) and allowed to reach confluence before the addition of stimuli. An epithelial volt-ohm meter (EVOM; World Precision Instruments, Sarasota, FL) was used to determine TEER values as described previously [20]. All measures were performed in triplicate and normalized for membrane area.

Protein uptake was studied after incubation of TEC with 50 mg/ml FITC-conjugated human albumin at 37°C for 2h. After FITC-albumin challenge, TEC were washed with ice-cold 1x PBS and analyzed by FACS.

### Statistical Analysis

Data analysis was performed with SPSS (Inc. Chicago IL, vers. 20.0.0). Distribution of variables was assessed by skewness and kurtosis; continuous non-normal distributed data were described using the median and range (min—max) and analyzed with Mann-Whitney test; normal distributed variables were described with mean and standard deviation and analyzed with two-tails t-test or ANOVA depending on groups number. NGAL plasma levels were adjusted by stepwise logistic regression multivariate analysis for potential confounding variables. Proportion based analysis was performed with χ^2^ test. A p value <0.05 was considered significant.

## Results

### KT patients’ data analysis

#### DGF incidence and clinical parameters of the study group

**Fig 1:**
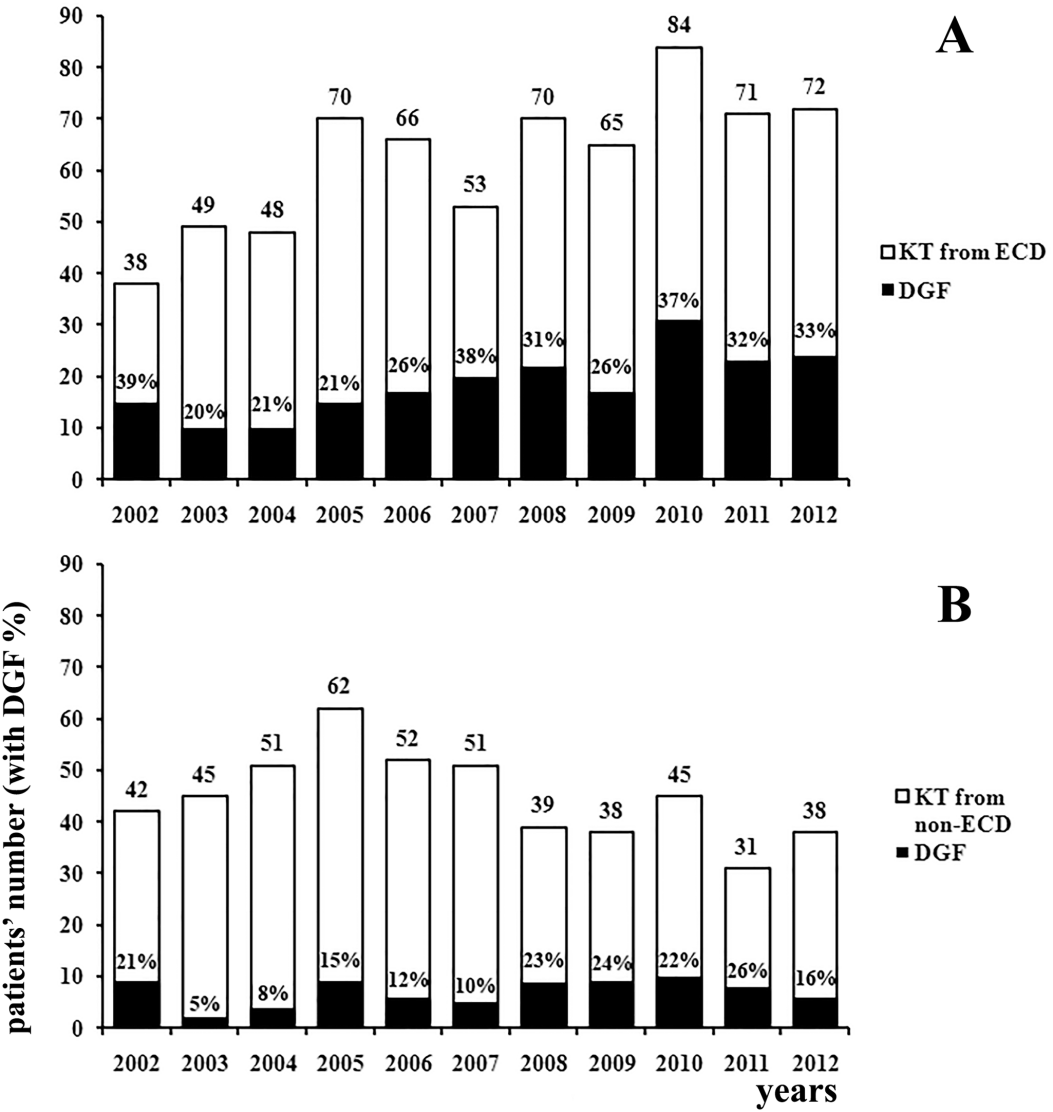
Number of KT performed (white bars) and relative DGF incidence (expressed as % of total KT in black bars) from 2002 to 2012 in KT from ECD (A) or non-ECD (B). DGF incidence in KT from ECD was significantly higher than in KT from non-ECD (p<0.0001). ECD: extended criteria donors; DGF: delayed graft function; KT: kidney transplantation.

**Fig 2:**
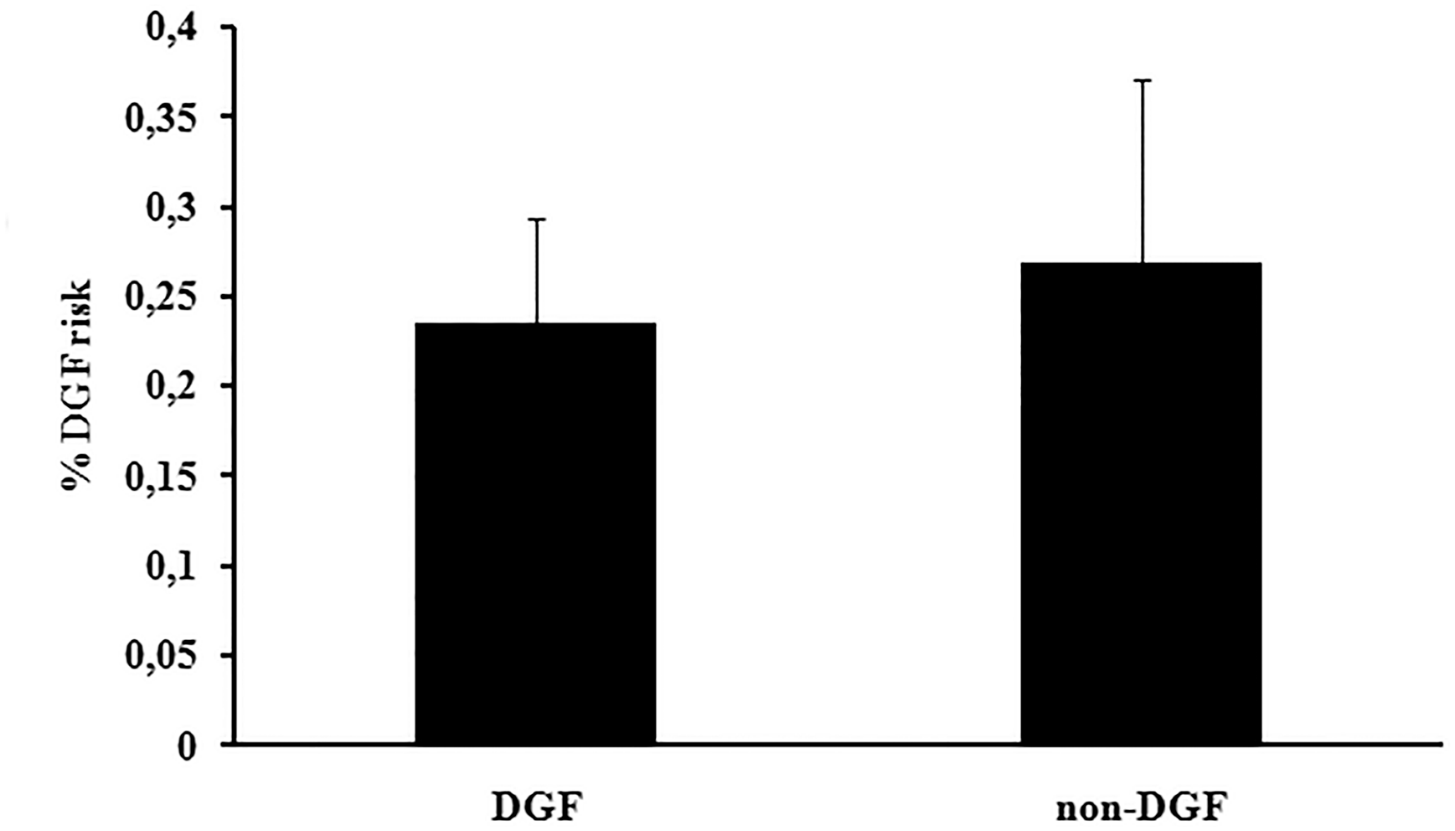
Percentage of DGF risk estimated by the prediction algorithm proposed by Irish et al. (DGF calculator). No significant differences between DGF and non-DGF KT patients were observed (p = 0.19, n = 50). DGF: delayed graft function.

On the basis of a retrospective study on 1180 KT performed in our centre between 2002 and 2012, we observed a significant overall higher incidence of DGF in KT recipients from ECD (ECD 29.7% vs. non ECD 15.6%; p<0.001) (Fig 1A and 1B). We previously analysed plasma NGAL levels in a first cohort of 25 kidney transplanted patients from ECD, observing that values >400 ng/ml were associated with DGF [21]. Therefore, we designed a prospective study in a second cohort of KT from ECD performed in 2013–2014: the incidence of DGF in this study group was 28% (14/50). The non-DGF group (n = 36) included 19 cases of SGF (53%) and 17 cases of IGF (47%); two patients developed acute rejection before hospital discharge, one patient of the DGF group and one of the IGF group. Comparison of donor-, recipient- and KT-associated variables between the studied group (n = 50) and all other KT from ECD performed between 2013 and 2014 (n = 93) did not show any significant differences. For this reason, we considered our study group as a representative sample of the whole KT population from ECD in our center (Table 1). We did not observe any significant difference in DGF vs. non-DGF group regarding donor and recipient age and co-morbidities, CIT, HLA-mismatches and donor pre-transplant Karpinski biopsy score (Table 2). We also applied the risk prediction model for DGF proposed by Irish et al. Values obtained by using the individual risk calculator were not significantly different between the DGF and the non-DGF group (DGF 0.23±0.06 vs. non-DGF 0.27±0.10; p = 0.2) (Fig 2).

**Table 1:** Comparison between study group and other patients underwent ECD KT between 2013 and 2014.

	*Study Group (n = 50)*	*Other ECD KT (2013–2014) (n = 93)*	*p*
***Donors’ variables***			
*Age (years)*	*65*.*8 (SD = 68*.*7)*	*67*.*1 (SD = 8*.*91)*	*0*.*8*
*BMI (kg/m2)*	*25*.*4 (SD = 3*.*1)*	*25*.*5 (SD = 3*.*25)*	*0*.*81*
*eGFR (ml/min)*	*91*.*2 (SD = 25*.*3)*	*88*.*1 (SD = 8*.*9)*	*0*.*5*
*sCr (mg/dl)*	*0*.*78 (SD = 0*.*32)*	*0*.*82 (SD = 0*.*27)*	*0*.*6*
*Hypertension*	*64% (n = 32)*	*58% (n = 54)*	*0*.*54*
*Diabetes*	*10% (n = 5)*	*7*.*5% (n = 7)*	*0*.*62*
*Cause of death*	*(n = 48/50)*	*(n = 93/93)*	
* - traumatic*	*32% (n = 16)*	*29% (n = 27)*	*0*.*5*
* - vascular*	*68% (n = 32)*	*71% n = (66)*	
***Recipients’ variables***			
*Age (years)*	*56*.*7 (SD = 9*.*3)*	*58*.*9 (SD = 9*.*6)*	*0*.*22*
*BMI (kg/m2)*	*24*.*2 (SD = 3*.*7)*	*24*.*6 (SD3*.*5)*	*0*.*51*
*Hypertension*	*71*.*4% (n = 35)*	*63*.*4% (n = 59)*	*0*.*53*
*Diabetes*	*18% (n = 9)*	*14% (n = 13)*	*0*.*56*
*Dialysis duration (months)*	*50*.*1 (SD = 62*.*14)*	*45*.*4 (SD = 31*.*6)*	*0*.*21* *
*Dialysis type*			
* - hemodialysis*	*78% (n = 39)*	*63% (n = 59)*	*0*,*07*
* - peritoneal*	*22% (n = 11)*	*37% (n = 24)*	
*PRA %*	*3*.*5 (SD = 13*.*1)*	*4*.*9 (SD = 14*.*5)*	*0*.*51*
*Gender*			
* - male*	*42% (n = 21)*	*40% (n = 37)*	*0*,*36*
* - female*	*58% (n = 29)*	*60% (n = 56)*	
*Previous KT*	*10% (n = 5)*	*13% (n = 12)*	*0*,*042*
*Discharge sCr (mg/dl)*	*1*.*93 (SD = 0*.*43)*	*1*.*97 (SD = 0*.*49)*	*0*.*69*
*Discharge pto (g/day)*	*0*.*57 (SD = 0*.*6)*	*0*.*51 (SD = 0*.*44)*	*0*.*57*
*Nephropathy*	*(n = 49/50)*	*(n = 93/93)*	
* - glomerular*	*20% (n = 10)*	*27% (n = 24)*	*0*.*52*
* - tubule-interstitial*	*12% (n = 6)*	*14% (n = 13)*	
* - vascular*	*14% (n = 7)*	*24*.*7% (n = 23)*	
* - diabetes*	*8% (n = 4)*	*3*.*2% (n = 4)*	
* - PKD*	*28% (n = 14)*	*15% (n = 14)*	
* - Others*	*16% (n = 8)*	*16*.*1% (n = 15)*	
***Transplants’ variables***			
*DGF (%)*	*28%(n = 14)*	*30% (n = 28)*	*0*.*51*
*Cold ischemia time (h)*	*16*.*6 (SD = 3*.*72)*	*17*.*7 (SD = 5*.*2)*	*0*.*15*
*HLA MM*	*(n = 47/50)*	*(n = 89/93)*	
* - 0*	*0% (n = 0)*	*0*.*01% (n = 1)*	*0*.*99*
* - 1*	*4% (n = 2)*	*5*.*3% (n = 5)*	
* - 2*	*14% (n = 7)*	*18*.*2% (n = 17)*	
* - 3*	*26% (n = 12)*	*25*.*8% (n = 24)*	
* - 4*	*40% (n = 20)*	*36*.*5% (n = 34)*	
* - 5*	*12% (n = 6)*	*8*.*6% (n = 8)*	
* - 6*	*0% (n = 0)*	*0% (n = 0)*	
*Biopsy Score*	*(n = 48/50)*	*(n = 82/93)*	
* - 0*	*6% (n = 3)*	*3*.*2% (n = 3)*	*0*.*83*
* - 1*	*10% (n = 5)*	*8*.*6% (n = 8)*	
* - 2*	*42% (n = 21)*	*33% (n = 31)*	
* - 3*	*34% (n = 17)*	*29% (n = 27)*	
* - 4*	*4% (n = 2)*	*10*.*7% (n = 10)*	
* - >4*	*0% (n = 0)*	*3*.*2% (n = 3)*	

Analysis of clinical variables related to donors, recipients and transplant procedures in the study group of KT from ECD (n = 50) vs. all KT from ECD (n = 93) performed in our center from 2013 to 2014. No significant differences between the two groups were observed. eGFR: estimated glomerular filtration rate; BMI: body mass index; DGF: delayed graft function; HLA MM: HLA-mismatches; KT: kidney transplant; N-DGF: not delayed graft function; PRA: panel reactive antibodies; pto: 24h proteinuria; sCr: serum creatinine; SD: standard deviation. The p values were calculated by t-student test for continues variables, by χ^2^ test for other variables

*variable “dialysis duration” has not a normal distribution, thus the “p-value” was calculated with Mann-Whitney test.

**Table 2:** Comparison between DGF and non-DGF groups.

Study Group (n = 50)	DGF (n = 14)	non-DGF (n = 36)	P
***Donors’ variables***			
*Age (years)*	*64*.*5 (SD = 8*.*5)*	*67*.*5 (SD = 7*.*25)*	*0*.*24*
*BMI (kg/m2)*	*26*.*3 (SD = 1*.*42)*	*25*.*2 (SD = 3*.*43)*	*0*.*985*
*eGFR (ml/min)*	*97*.*5 (SD = 25*.*3)*	*89*.*1 (SD = 26*.*6)*	*0*.*332*
*sCr (mg/dl)*	*0*.*76 (SD = 0*.*23)*	*0*.*79 (SD = 0*.*38)*	*0*.*722*
*Hypertension*	*57*.*1% (n = 8)*	*66*.*7% (n = 24)*	*0*.*67*
*Diabetes*	*14*.*2%% (n = 2)*	*8*.*3% (n = 3)*	*0*.*19*
*Death (n = 48)*			
* - traumatic*	*50% (n = 6)*	*27*.*8% (n = 10)*	*0*.*18*
* - vascular*	*50% (n = 6)*	*72*.*7% (n = 26)*	
***Recipients’ variables***			
*Age (years)*	*54*.*5 (SD = 10*.*9)*	*57*.*5 (SD = 8*.*9)*	*0*.*333*
*BMI (kg/m2)*	*24*.*1 (SD = 3*.*59)*	*24*.*3 (SD = 3*.*77)*	*0*.*888*
*Hypertension*	*57*.*1% (n = 8)*	*75% (n = 27)*	*0*.*67*
*Diabetes*	*7*.*1% (n = 1)*	*21*.*6% (n = 8)*	*0*.*317*
*Dialysis duration (months)*	*81*.*9(SD = 110*.*2)*	*26*.*8 (SD = 39*.*9)*	*0*.*59* *
*Dialysis type*			
* - hemodialysis*	*91*.*7%(n = 11)*	*75% (n = 27)*	*0*.*23*
* - peritoneal*	*8*.*3%(n = 1)*	*25% (n = 9)*	
*PRA %*	*3*.*7 (SD = 14*.*7)*	*2*.*27 (SD = 6*.*32)*	*0*.*63*
*Gender*			
* - male*	*57*,*1% (n = 8)*	*36*.*1% (n = 13)*	*0*.*532*
* - female*	*42*.*9% (n = 6)*	*63*.*9% (n = 23)*	
*Previous KT*	*14*.*3% (n = 2)*	*8*.*3% (n = 3)*	*0*.*423*
*Discharge sCr (mg/dl)*	*2*.*08 (SD = 0*.*36)*	*1*.*89 (SD = 0*.*45)*	*0*.*157*
*Discharge pto (g/day)*	*0*.*56 (SD = 0*.*37)*	*0*.*58 (SD = 0*.*68)*	*0*.*726*
*Nephropathy*	*(n = 13/14)*	*(n = 36/36)*	
* - glomerular*	*33*.*3% (n = 4)*	*16*.*2% (n = 6)*	*0*.*651*
* - tubule-interstitial*	*16*.*7% (n = 2)*	*10*.*8% (n = 4)*	
* - vascular*	*8*.*3% (n = 1)*	*16*.*2% (n = 6)*	
* - diabetes*	*0% (n = 0)*	*10*.*8% (n = 4)*	
* - PKD*	*25% (n = 4)*	*29*.*7% (n = 10)*	
* - others*	*15*.*4% (n = 2)*	*16*.*2% (n = 6)*	
***Transplants’ variables***			
*Cold ischemia time (h)*	*16*.*42 (SD = 3*.*95)*	*17*.*2 (SD = 2*.*95)*	*0*.*457*
*HLA MM*	*(n = 11/14)*	*(n = 36/36)*	
* - 0*	*0% (n = 0)*	*0% (n = 0)*	*0*.*342*
* - 1*	*0% (n = 0)*	*5*.*5% (n = 2)*	
* - 2*	*27*.*2%(n = 3)*	*11*.*1% (n = 4)*	
* - 3*	*36*.*4% (n = 4)*	*22*.*2% (n = 8)*	
* - 4*	*36*.*4% (n = 4)*	*44*.*4% (n = 16)*	
* - 5*	*0% (n = 0)*	*16*.*6% (n = 6)*	
* - 6*	*0% (n = 0)*	*0% (n = 0)*	
*Biopsy Score*	*(n = 12/14)*	*(n = 36/36)*	
* - 0*	*8*.*3% (n = 1)*	*5*.*5% (n = 2)*	*0*.*831*
* - 1*	*8*.*3% (n = 1)*	*11*.*1% (n = 4)*	
* - 2*	*50% (n = 6)*	*41*.*7% (n = 15)*	
* - 3*	*25% (n = 3)*	*38*.*9% (n = 14)*	
* -* ≥ *4*	*8*.*3% (n = 1)*	*2*.*3% (n = 1)*	

Analysis of clinical variables related to donors, recipients and transplant procedures in the study group of KT from ECD (n = 50) subdivided between patients with DGF (n = 13) and non-DGF (n = 37). No significant differences between DGF and non-DGF group were observed. eGFR: estimated glomerular filtration rate; BMI: body mass index; DGF: delayed graft function; HLA MM: HLA-mismatches; KT: kidney transplant; N-DGF: not delayed graft function; PRA: panel reactive antibodies; pto: 24h proteinuria; sCr: serum creatinine; SD: standard deviation. The p values were calculated by t-student test for continues variables, by χ^2^ test for other variables

*variable “dialysis duration” has not a normal distribution, thus the “p-value” was calculated with Mann-Whitney test.

#### NGAL values predicted graft recovery and increased after Tac administration

**Fig 3:**
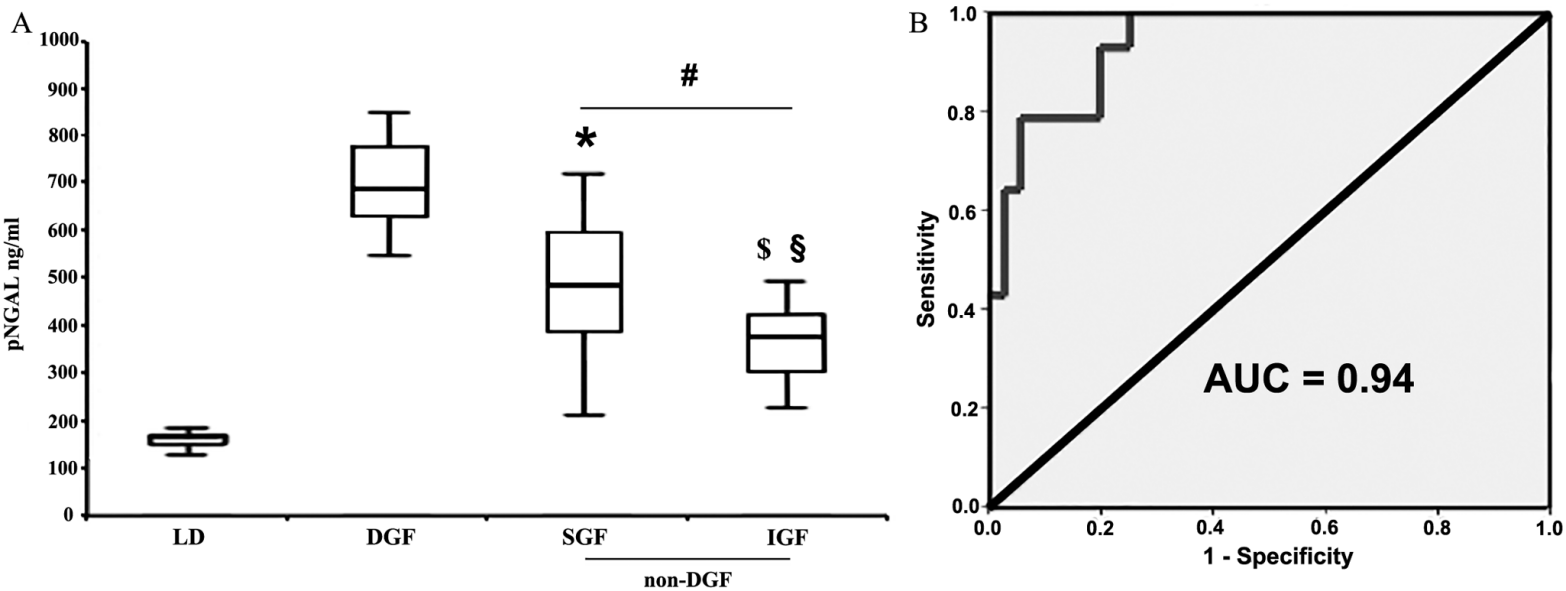
Evaluation of plasma NGAL levels at day 1 after KT in patients subdivided on the basis of graft function (DGF: Delayed Graft Function; SGF: Slow Graft Function; IGF: Immediate Graft Function). (A) NGAL was significantly higher in DGF (662.7 ±97.2 ng/ml, n = 14) vs. non-DGF patients (SGF + IGF: 379.7±139.7 ng/ml, n = 36; #p<0.00001). A statistical significance was also observed comparing DGF vs SGF (444.1±149.5 ng/ml, n = 19; *p<0.001) or IGF (307.8±101.8 ng/ml, n = 17; $p<0.00001) and SGF vs IGF (§p<0.01). NGAL levels of KT from living donors (LD) were used as control. (B) The Area Under the ROC curve (AUC) of NGAL as biomarker of DGF at day 1 post-KT was 0.94.

**Fig 4:**
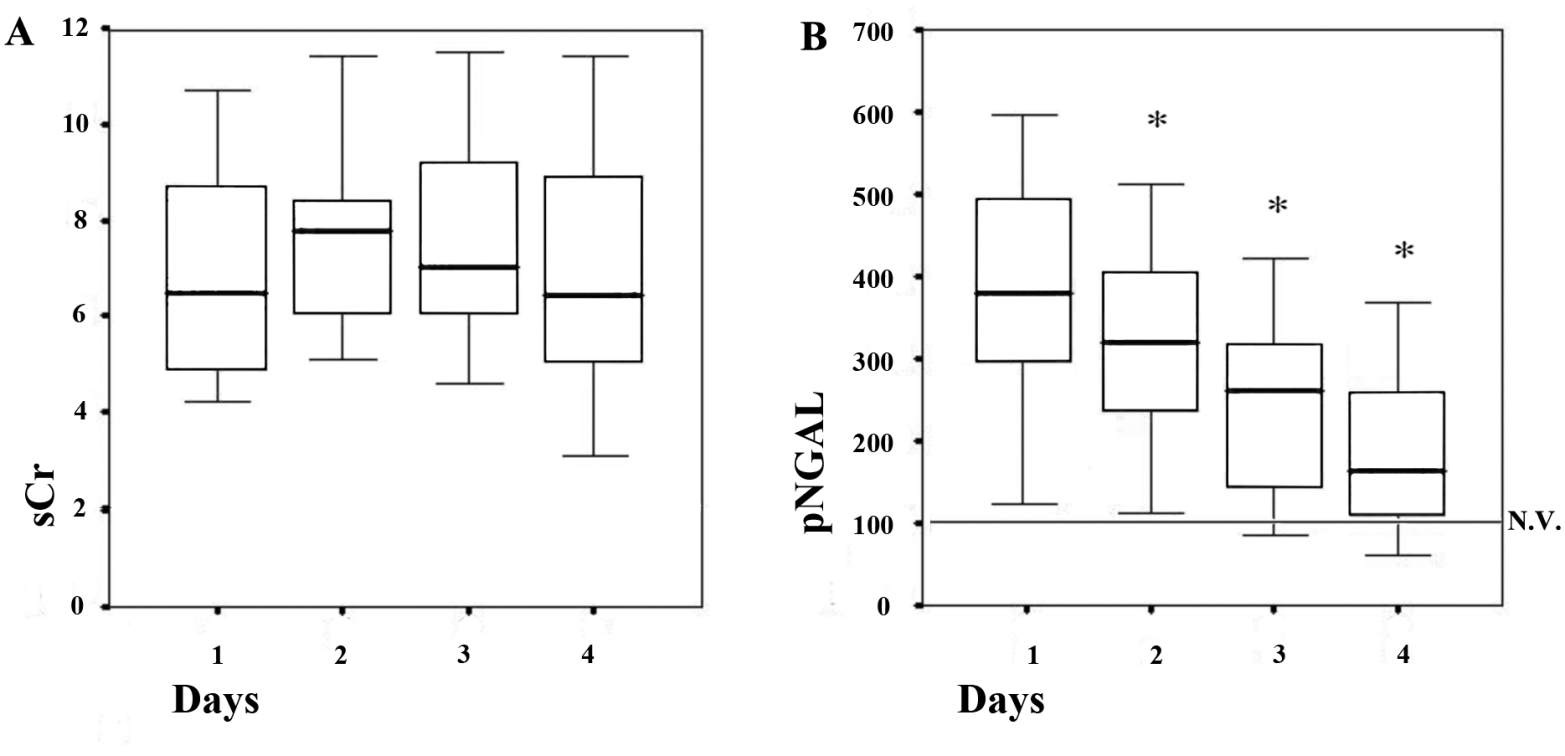
Evaluation of serum creatinine (sCr in A) and plasma NGAL (pNGAL in B) in the first 4 consecutive days after KT in non-DGF patients (n = 36). NGAL significantly decreased starting from day 2 to day 4 after KT reaching a value near to normal levels (100 ng/ml). Each value of pNGAL was significantly lower in comparison to the previous day (*p<0.05). By contrast, sCr remained stable or even increased (p>0.05).

**Fig 5:**
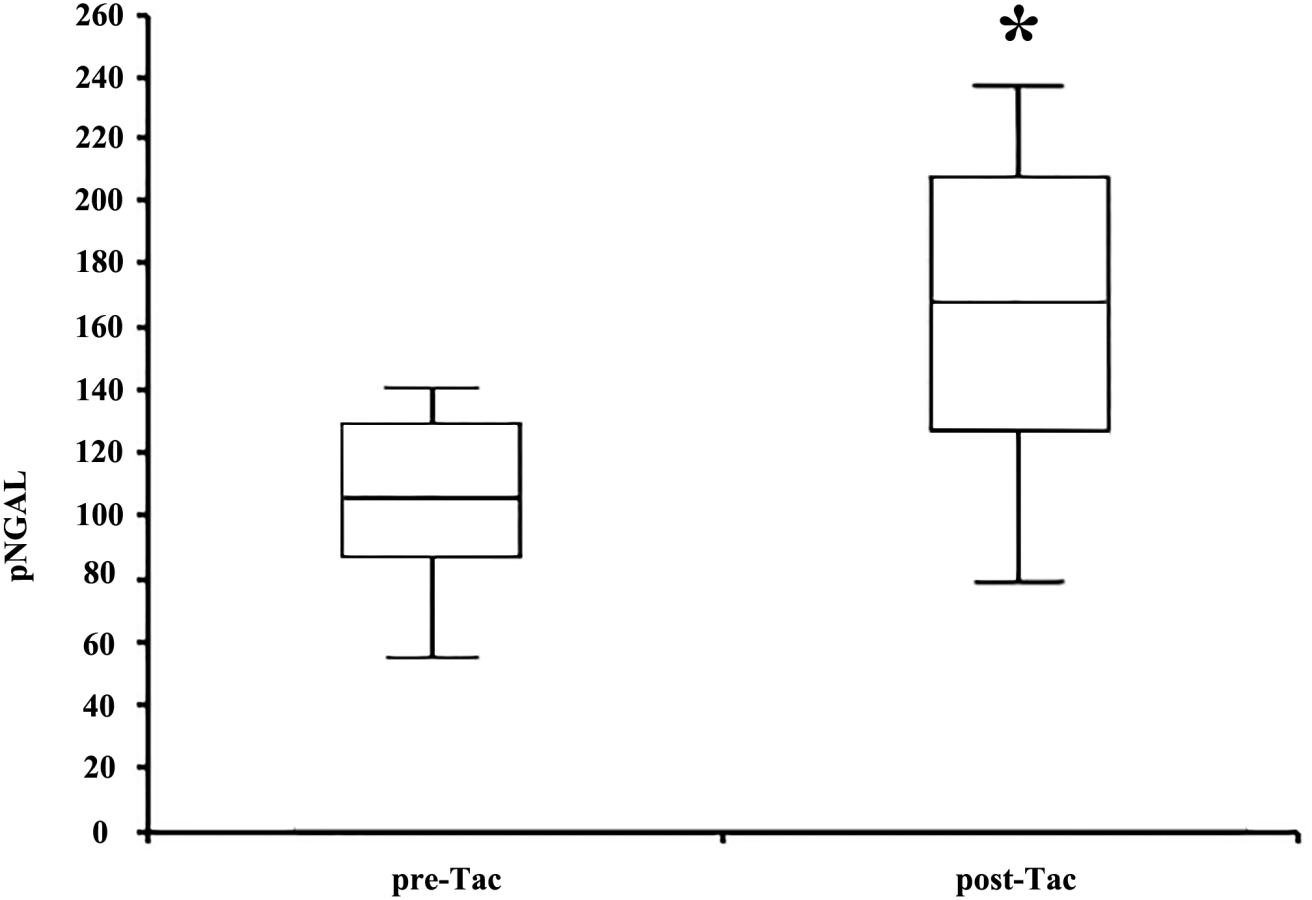
Evaluation of plasma NGAL levels before and after Tacrolimus (Tac) introduction. NGAL was significantly higher after Tac administration (post-Tac) than before drug introduction (pre-Tac) (*p<0.001, n = 50).

Mean NGAL levels in the DGF group were 662.7±97,2 ng/ml vs. 379.7±139.7 ng/ml in the non-DGF group (p< 0.001) (Fig 3A). Pre-transplant levels of NGAL of all enrolled patients were similar to those detected in the DGF group at day 1 post-transplantation (632±84ng/ml; p = 0,25). The difference between DGF and non-DGF group was still significant in multivariate analysis after correcting NGAL values for day 1 serum creatinine, donor and recipient age, cold ischemia time, donor diabetes and hypertension or histological score (p<0,01). Using the ROC curve, we identified a level of NGAL of 532 ng/ml as the more sensitive and specific value to distinguish between DGF and non-DGF (sensitivity 90.9%; specificity 80.6%, AUC 0.94, Fig 3B). In addition, in the non-DGF group NGAL was able to distinguish between SGF and IGF with statistical significance. In the SGF group mean NGAL levels were 444.1±149.5 ng/ml vs. 307.8±84.5 ng/ml in the IGF group (p<0.001). In the control population of 10 KT from living donors (with very short CIT), mean NGAL levels were 67.3±25 ng/ml (Fig 3A). In the non-DGF group, NGAL was further measured for 4 consecutive days after KT. In non-DGF patients NGAL showed an earlier decrease in comparison to serum creatinine that remained stable or even increased (Fig 4A and 4B). Furthermore, NGAL levels were evaluated in all enrolled patients the day before and the day after Tac administration. The average time from transplantation to CNI introduction was 11.04±4.86 days. Mean NGAL levels before and after Tac administration were respectively 107.9±37.66 ng/ml and 155.9±48.3 ng/ml (p< 0.001—Fig 5). By contrast, we did not find any significant difference between serum creatinine levels pre- and post-Tac introduction (2.33±0.22 vs 2.38±0.31; p = 0.4 –data not shown in figs).

### *In vitro* assays on TEC

#### Hypoxia and Tac increased NGAL expression in cultured human TEC

**Fig 6:**
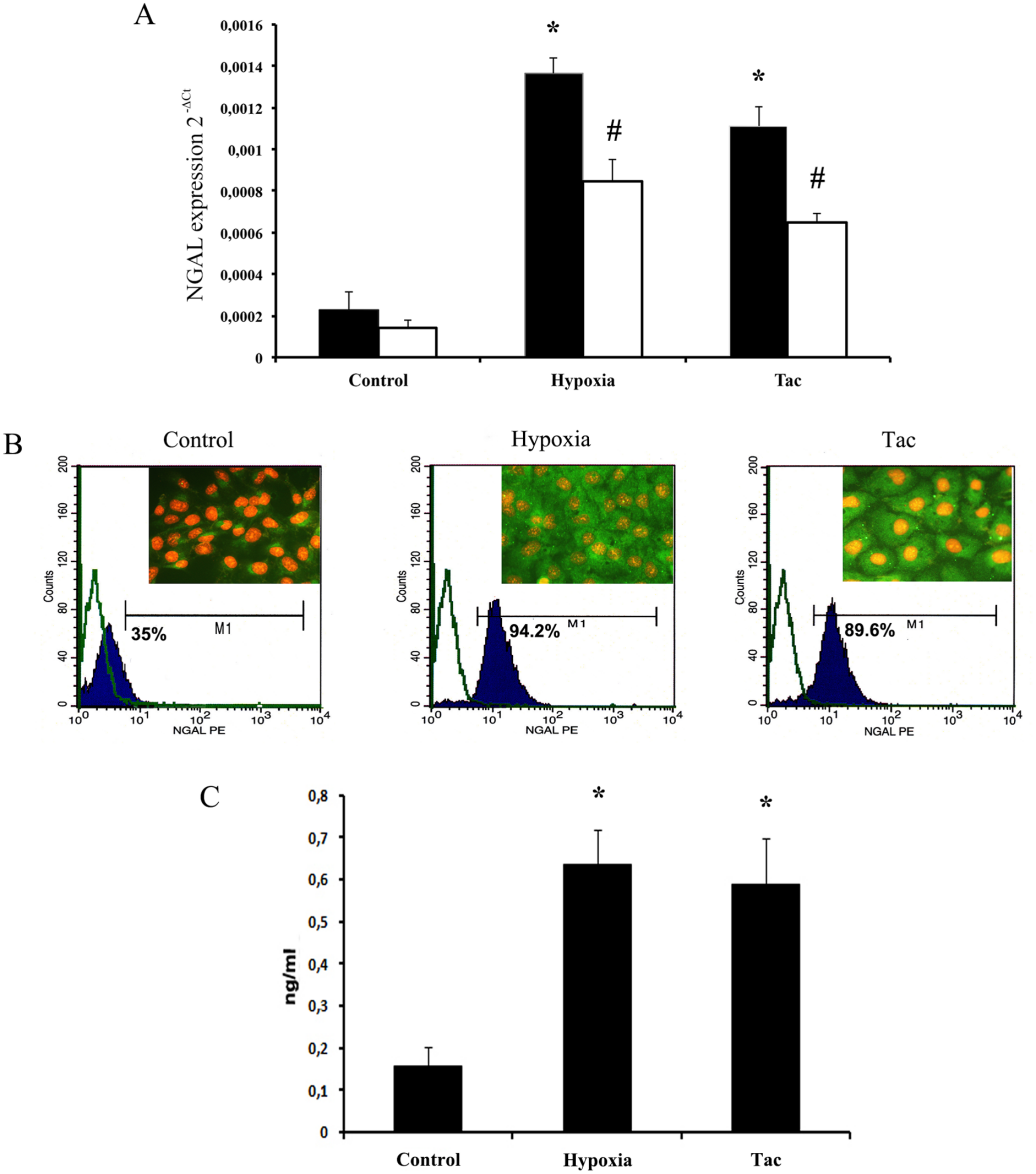
*In vitro* analysis of NGAL expression and release by human tubular epithelial cells (TEC) in different experimental conditions. Relative quantification analysis (A) of NGAL mRNA expression in TEC evaluated by qRT-PCR; mRNA expression of 3 different experiments was normalized for both GAPDH (black columns) and Beta-Actin (white columns) after calculating housekeeping gene variation (see also methods). Hypoxia and 20 ng/ml Tacrolimus (Tac) significantly increased NGAL mRNA expression when compared to basal culture condition (Control, *p<0.001, #p<0.001). FACS (B) and immunofluorescence (B inserts) analysis confirmed Hypoxia- and Tac-induced NGAL production. For FACS, Kolmogorov-Smirnov statistical analysis was performed; control isotype antibody is represented by white plots, NGAL staining by black plots. For immunofluorescence analysis, nuclei were counterstained with 1μg/ml propidium iodide, original magnification was x200. (C) ELISA analysis of NGAL released from TEC. Hypoxia and Tacrolimus (Tac) significantly increased NGAL release in TEC supernatants (*p<0.01).

By using qRT-PCR, we detected a significant increase of NGAL mRNA expression in TEC after 24h culture in hypoxic conditions and after 24h stimulation with 20 ng/ml Tac (p<0.001—Fig 6A, amplification curve in S2 Fig). No significant differences in the housekeeping genes Beta-Actin and GAPDH were observed in both hypoxic and Tac-stimulated TEC (data not shown).

The percentage of TEC producing NGAL measured by FACS showed an increase from 35% (basal condition) to 94.2% and 89.6% after 24h of stimulation in hypoxic conditions and with Tac, respectively (Fig 6B). NGAL was also measured by ELISA in TEC supernatants: hypoxia induced a 4.05±0.53 fold increase (p<0.01), while 24h stimulation with Tac induced a 3.74±0.71 fold increase (p<0.01—Fig 6C).

#### NGAL protected TEC from hypoxia- and Tac-induced apoptosis and functional alterations

**Fig 7:**
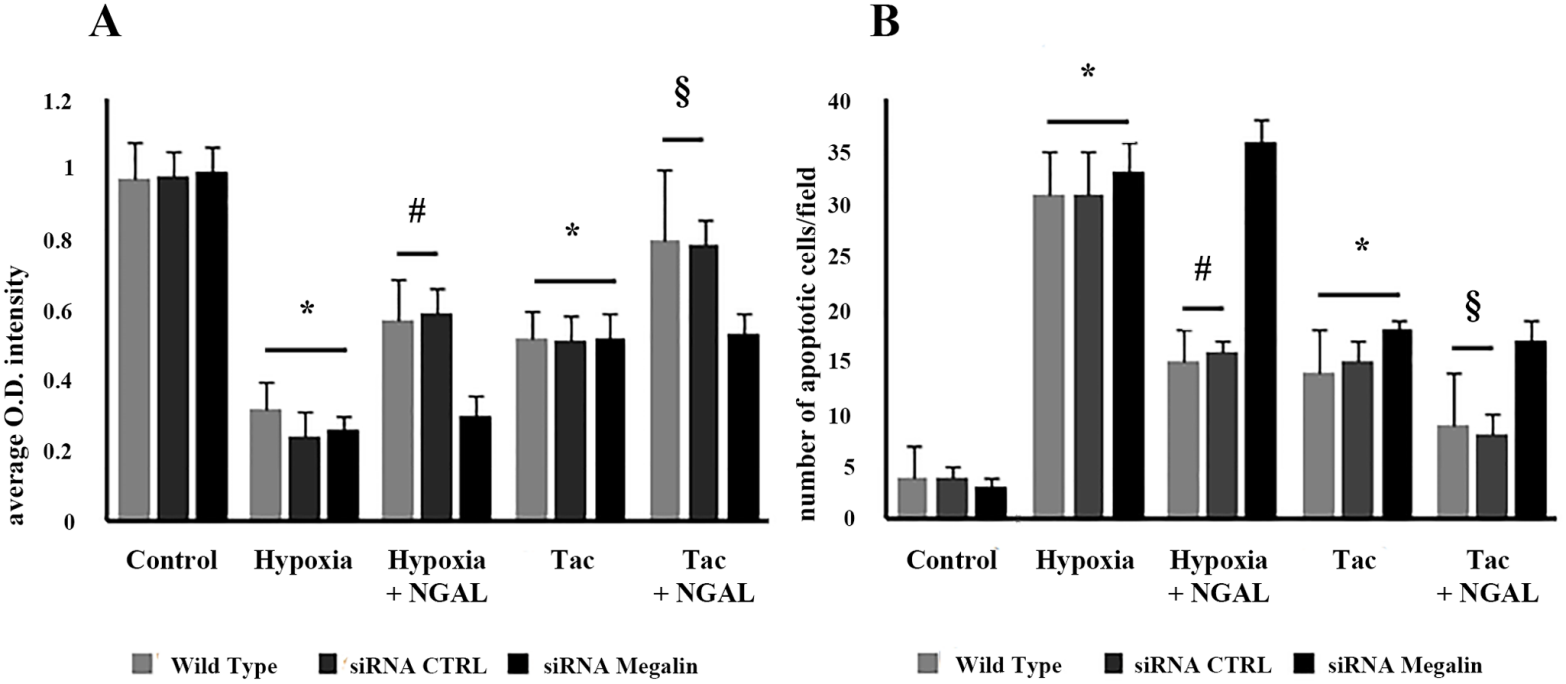
*In vitro* analysis of TEC cytotoxicity (XTT assay in A) and apoptosis (TUNEL assay in B) in different experimental conditions. Hypoxia and Tac (20 ng/ml) significantly increased cytotoxicity (A, *p<0.01) and apoptosis (B, *p<0.01) in wild type TEC (white bars). NGAL (500 ng/ml) significantly decreased both hypoxia- and Tac-induced TEC cytotoxicity (A: #p<0.01, §p<0.05); and apoptosis (B: #p<0.01, §p<0.05). This effect was not observed after megalin knock-down by siRNA (siRNA Megalin in black bars) but not by transfection with an irrelevant siRNA (siRNA CTRL in gray bars).

**Fig 8:**
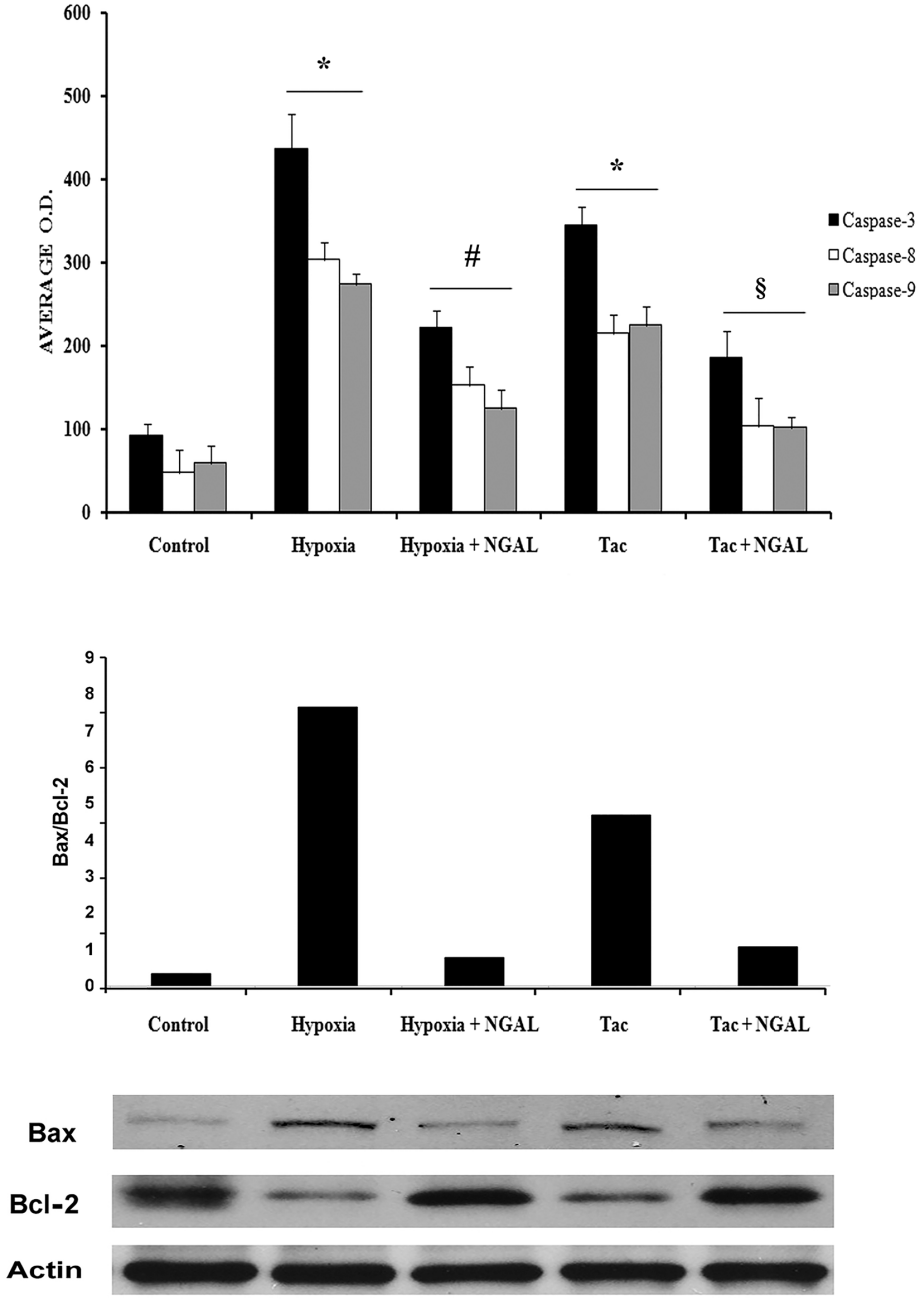
(A) ELISA for caspase-3, -8 and -9 activity in TEC cultured in different experimental condition. Hypoxia and Tac (20 ng/ml) significantly increased caspase-3, -8, -9 activity (*p<0.05). NGAL (500 ng/ml) significantly decreased both hypoxia- (#p<0.05) and Tac-induced (§p<0.05) caspase-3, -8, -9 activity. (B) Western Blot analysis of Bax/Bcl2 and relative quantitative analysis expressed as Bax/Bcl2 ratio. Hypoxia and Tac (20 ng/ml) increased Bax/Bcl2 ratio. NGAL (500 ng/ml) decreased both Bax/Bcl2 ratio. Values of Bax and Bcl2 were previously normalized for Beta-Actin expression. Three different experiments were performed with similar results.

Culture in hypoxic conditions or incubation with 20 ng/ml Tac decreased viability and increased apoptosis of TEC (p<0.001) (Fig 7). NGAL (500 ng/ml) significantly reduced hypoxia- and Tac-induced cytotoxicity and apoptosis (Fig 7A and 7B, respectively). The specific role of NGAL was confirmed by experiments with TEC previously engineered by specific siRNA to knock-down megalin, the NGAL receptor. Transfection of TEC with siRNA megalin (qRT-PCR and western blot in S3 Fig) significantly reduced the anti-cytotoxic and anti-apoptotic effect of NGAL. These results were not observed when TEC were transfected with an irrelevant control siRNA (Fig 7). Moreover, we found that recombinant NGAL decreased the expression of caspase-3, -8, -9 (Fig 8A) and the Bax/Bcl-2 ratio in TEC cultured in hypoxia or with Tac (Fig 8B and 8C). These results suggest a putative role of NGAL in the inhibition of both death receptor and mitochondrial pathways of TEC apoptosis.

**Fig 9:**
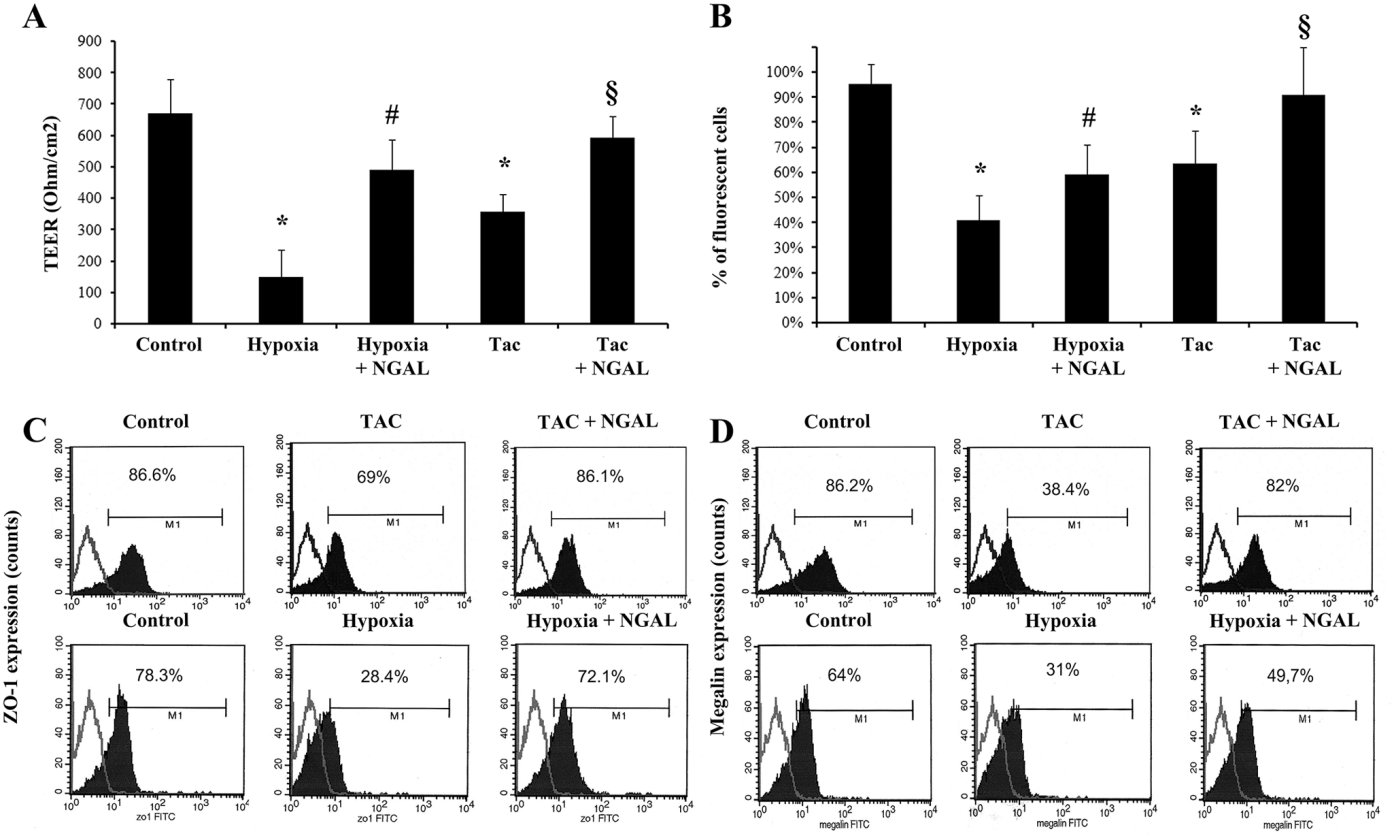
TEC functional assays after hypoxia or Tac. (A) Analysis of TEC polarity assessed by trans-epithelial electrical resistance (TEER) in different experimental conditions. Hypoxia and Tac (20 ng/ml) significantly decreased TEER (*p<0.05). NGAL (500 ng/ml) significantly increased TEER in both hypoxia- (#p<0.05) and Tac-treated (§p<0.05) TEC. (B) Analysis of uptake of FITC-conjugated albumin by TEC in different experimental conditions. Hypoxia and Tac (20 ng/ml) significantly decreased albumin uptake (*p<0.05). NGAL (500 ng/ml) significantly increased albumin uptake in both hypoxia- (#p<0.05) and Tac-treated (§p<0.05) TEC. (C-D) FACS analysis of the tight junction molecule ZO-1 (C) and of the endocytic receptor megalin (D) in TEC cultured in different experimental conditions. Hypoxia and Tac significantly decreased ZO-1 and megalin expression. NGAL (500 ng/ml) up-regulated ZO-1 and megalin in both hypoxia- and Tac-treated TEC. Kolomogorov Smirnov statistical analysis was performed in three different experiments with similar results.

Moreover, hypoxia and Tac induced in TEC loss of cell polarity assessed by trans-epithelial electrical resistance (TEER in Fig 9A), reduction of albumin uptake (Fig 9B), down-regulation of the tight junction protein ZO-1 (Fig 9C) and of the endocytic receptor megalin (Fig 9D). All these functional changes were significantly decreased when 500ng/ml recombinant NGAL was added to hypoxic and Tac-treated TEC.

## Discussion

Previous studies showed a correlation between plasma and urine NGAL levels and DGF in KT [21]. However, none of these studies were performed in KT from ECD and using CNI-sparing protocols in the early phase after the procedure, with the impossibility to discriminate between ischemia-reperfusion injury and drug nephrotoxicity. We herein showed that the plasma level of NGAL in the first 24h after KT from ECD without CNI therapy is an accurate biomarker for DGF identification with sensitivity 92.9%, specificity 80.6% and AUC 0.94. Moreover, we found that in non-DGF patients, NGAL was able to distinguish between SGF and IGF with statistical significance. NGAL was more sensitive than serum creatinine to predict functional recovery. Indeed, we observed that NGAL levels decreased more rapidly than serum creatinine, suggesting re-establishment of both glomerular filtration and tubular re-absorption. Therefore, these results suggest a potential use of NGAL determination for introducing CNI therapy even though serum creatinine levels have not reached 2.5 mg/dl yet.

Another novelty of the present study was that NGAL offered a better monitoring of renal response to Tac administration in comparison to serum creatinine. The use of NGAL as indicator of CNI toxicity was recently proposed in cyclosporine-treated steroid-dependent nephrotic children [22] but not in the context of KT. Furthermore, the *in vitro* studies provided information on the functional role of NGAL indicating that its expression and release by human TEC may have anti-apoptotic and regenerative effects.

Despite several causes are known to favor DGF, ischemia-reperfusion injury, CNI nephrotoxicity and use of organs from ECD are recognized as main determinant factors [23,24]. Our retrospective analysis confirmed a significant higher incidence of DGF in KT from ECD. Indeed, the mean incidence of DGF in our study group was 26% despite the adoption of CNI sparing protocols in the early phase after KT and to the policy of minimization of ischemia-reperfusion injury (mean CIT of 16.42h).

The clinical relevance of DGF is associated with an increased risk of T cell-mediated rejection for the enhanced immunogenicity of TEC after ischemia-reperfusion injury due to exposure of hidden antigens [25,26]. In addition, Fuquay et al. recently demonstrated that renal ischemia-reperfusion injury may amplify the humoral immune response due to foreign antigen injection in mice, suggesting that DGF may also increase the risk of antibody-mediated rejection [27]. The association between ischemic and immune-mediated damage may lead to an early deterioration of graft function that may be ascribed to development of aberrant mechanisms of tubular repair and regeneration, finally leading to epithelial-to-mesenchymal transition and tissue fibrosis [28].

On the basis of the previous considerations, the development of early, accurate and noninvasive biomarkers of graft function may facilitate DGF diagnosis. Several molecules identified as indicators of acute renal dysfunction in native kidneys have been proposed for the detection of DGF in KT. Among these, urine levels of IL-18 and NGAL have been shown to identify DGF with acceptable statistical relevance [29]. However, in the majority of cases the low urine output in the first hours after KT and the need of ratio with urine creatinine may generate some interpretative bias. For these reasons, we decided to study plasma levels of NGAL, a 25KDa protein belonging to the lipocalin family. Lipocalins, together with avidins and fatty acid binding proteins, constitute the calycin superfamily, a class of low-molecular weight proteins characterized by a hydrophobic binding site [30]. The main biological function of NGAL is the transport of iron through small molecules called siderophora. During infections, neutrophils release NGAL to regain iron chelated by bacterial siderophora with a consequent bacteriostatic activity. Moreover, during embryogenesis and tissue damage NGAL is known to promote differentiation and regeneration processes by iron delivery. The plasma pool of NGAL is mainly composed by the quote released by neutrophils, monocytes and hepatocytes that is freely filtered by glomeruli for its low molecular weight and re-adsorbed by proximal TEC through a megalin-dependent mechanism [31]. The urine pool of NGAL is composed by the quote not re-uptaken by proximal TEC and by a further quote produced in the distal part of the nephron as a consequence of ischemic or toxic tubular injury [31]. Moreover, plasma NGAL levels are strongly influenced by the presence of a chronic impairment of renal function and of an inflammatory state [32]. Our experience confirmed this observation: pre-transplant NGAL levels were high and comparable to those detected in the DGF group.

NGAL production has been shown to be up-regulated in different experimental models of TEC injury as well as in AKI patients. However, the biological activity of NGAL on TEC still remains controversial. Following nephron reduction, NGAL knock-out mice presented a significant decrease of proliferative renal lesions in respect to wild type animals [33]. NGAL expression was up-regulated after EGFR activation through a mechanism dependent on HIF-1α modulation. These results were confirmed in human CKD in which NGAL was particularly increased in patients with a rapid progression toward end stage renal failure. Taken together, these findings suggest that NGAL may be a reliable marker of tubular de-differentiation playing a role in the aberrant mechanisms of tissue repair and promotion of fibrosis already reported for other AKI biomarkers such as KIM-1 [34]. By contrast, other studies demonstrated that NGAL may act as a growth and differentiation factor in several cell types including renal epithelial cells [35]. This effect may be ascribed to the up-regulation of heme oxygenase-1 and to the consequent inhibition of cell death induced by oxidative stress [7,36]. In the present study, we observed that NGAL significantly reduced hypoxia-associated *in vitro* human TEC apoptosis through the inhibition of caspase activation and the reduction of Bax/Bcl-2 ratio. These results are in accordance with previous studies showing that NGAL inhibited apoptosis and bax/caspase-3 expression in a rat experimental model of renal artery ligation [10]. We also found that NGAL exerted on Tac-treated TEC a proliferative and anti-apoptotic effect similar to that observed in hypoxic condition, suggesting a putative beneficial role of this iron carrier molecule on both ischemic and nephrotoxic injury. Furthermore, NGAL preserved cell polarity and function in hypoxic and Tac-treated TEC as assessed by TEER, albumin uptake and staining for the endocytic receptor megalin and for the tight junction protein ZO-1. All these biological activities were observed *in vitro* using NGAL concentrations similar to those observed in KT patients. In addition, these effects were specific for NGAL, since siRNA-induced knock-down of megalin, the main NGAL receptor located on TEC surface, significantly abrogated its protective actions.

In conclusion, we demonstrated that NGAL is an early and accurate biomarker of graft function in KT from ECD. Moreover, NGAL is able to discriminate DGF from SGF or IGF with high sensitivity and specificity and it seems to be more sensitive than serum creatinine for monitoring Tac toxicity. Finally, our *in vitro* studies suggested that TEC may react to injurious stimuli triggering NGAL production via an autocrine loop aimed to sustain cell proliferation and inhibition of apoptosis associated with iron uptake.

## Supporting Information

S1 Dataset Clinical and experimental data. Dataset is organized in 9 sections; each section displays data of corresponding figs.
(XLSX)

S1 Fig *In vitro* analysis of cytotoxicity (XTT assay) of TEC cultured in hypoxic condition. Recombinant NGAL significantly increased hypoxic TEC viability in a dose-dependent manner starting from 100 ng/ml (p<0.001). The highest level of NGAL-induced TEC proliferation was observed using 500 ng/ml (p<0.001).
(TIF)

S2 Fig Representative amplification curve of NGAL mRNA expression by quantitative Real Time PCR (q-RT-PCR): in basal conditions (Control), in hypoxic conditions (Hypoxia) or with Tacrolimus (Tac). (TIF)

S3 Fig Quantification by qRT-PCR (A) and western blot analysis (B) of megalin in wild type TEC or in TEC transfected with 80 pM siRNA (siRNA megalin) or with 80 pM control siRNA (siRNA control). For qRT-PCR, megalin mRNA levels were normalized for the housekeeping gene Beta-Actin. For western blot analysis, megalin protein levels were normalized for Beta-Actin (Actin).
(TIF)

S1 File FACS Analysis and RNA Interference (Method). For FACS analysis, cells were detached with EDTA and stained for 1 h at 4°C with a primary antibody directed to human NGAL, Megalin, ZO-1 or with an irrelevant control antibody. After extensive washing, cells were incubated with Alexa Fluor—conjugated secondary antibodies for 45 min at 4°C where appropriate. All incubation periods were performed using a medium containing 0.25% BSA and 0.0016% sodium azide. At the end of staining, cells were newly washed, fixed in 1% paraformaldehyde, and subjected to FACS analysis (Becton Dickinson, Mountain View, CA). In selected experiments, TEC were seeded on six-well plates and Megalin siRNA, or relative control siRNA (80 pM) was introduced according to the manufacturer’s instructions (Santa Cruz Biotechnology). After 48 h, the effective suppression of specific mRNA and proteins was verified by RT-PCR, by WB and by citofluorimetric analysis (unpublished data). Subsequently, the cells were used to evaluate proliferation and apoptosis.
(DOCX)
