# Supplementary figures and images for: Neutrophil Gelatinase Associated Lipocalin Is an Early and Accurate Biomarker of Graft Function and Tissue Regeneration in Kidney Transplantation from Extended Criteria Donors
Source: PLoS One. 2015 Jun 30;10(6):e0129279. doi: 10.1371/journal.pone.0129279 (PMC4488380; doi:10.1371/journal.pone.0129279)

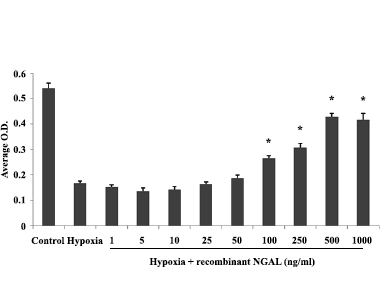

Supplement: S1 Fig — Recombinant NGAL significantly increased hypoxic TEC viability in a dose-dependent manner starting from 100 ng/ml (p<0.001). The highest level of NGAL-induced TEC proliferation was observed using 500 ng/ml (p<0.001). (TIF) [file pone.0129279.s002.tif]

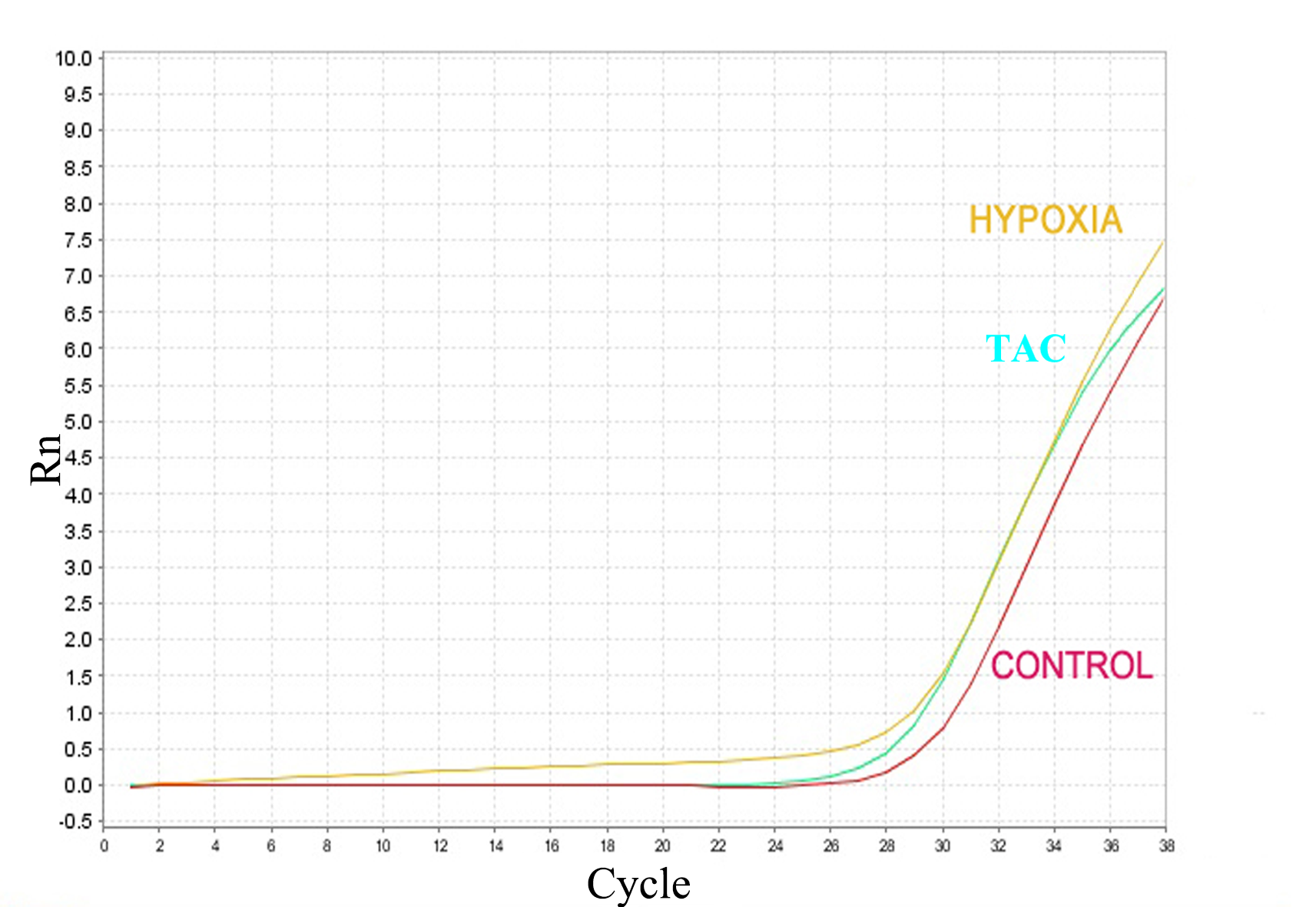

Supplement: S2 Fig — (TIF) [file pone.0129279.s003.tif]

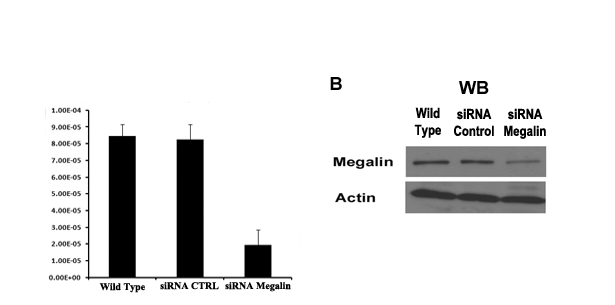

Supplement: S3 Fig — For qRT-PCR, megalin mRNA levels were normalized for the housekeeping gene Beta-Actin. For western blot analysis, megalin protein levels were normalized for Beta-Actin (Actin). (TIF) [file pone.0129279.s004.tif]
