# Supplementary material for: Neutrophil Gelatinase Associated Lipocalin Is an Early and Accurate Biomarker of Graft Function and Tissue Regeneration in Kidney Transplantation from Extended Criteria Donors
Source: PLoS One. 2015 Jun 30;10(6):e0129279. doi: 10.1371/journal.pone.0129279 (PMC4488380; doi:10.1371/journal.pone.0129279)
Supplement: S1 File — For FACS analysis, cells were detached with EDTA and stained for 1 h at 4°C with a primary antibody directed to human NGAL, Megalin, ZO-1 or with an irrelevant control antibody. After extensive washing, cells were incubated with Alexa Fluor—conjugated secondary antibodies for 45 min at 4°C where appropriate. All incubation periods were performed using a medium containing 0.25% BSA and 0.0016% sodium azide. At the end of staining, cells were newly washed, fixed in 1% paraformaldehyde, and subjected to FACS analysis (Becton Dickinson, Mountain View, CA). In selected experiments, TEC were seeded on six-well plates and Megalin siRNA, or relative control siRNA (80 pM) was introduced according to the manufacturer’s instructions (Santa Cruz Biotechnology). After 48 h, the effective suppression of specific mRNA and proteins was verified by RT-PCR, by WB and by citofluorimetric analysis (unpublished data). Subsequently, the cells were used to evaluate proliferation and apoptosis. (DOCX) [file pone.0129279.s005.docx]

**S1 File: FACS Analysis and RNA Interference (Method).** For FACS analysis, cells were detached with EDTA and stained for 1 h at 4°C with a primary antibody directed to human NGAL, Megalin, ZO-1 or with an irrelevant control antibody. After extensive washing, cells were incubated with Alexa Fluor– conjugated secondary antibodies for 45 min at 4°C where appropriate. All incubation periods were performed using a medium containing 0.25% BSA and 0.0016% sodium azide. At the end of staining, cells were newly washed, fixed in 1% paraformaldehyde, and subjected to FACS analysis (Becton Dickinson, Mountain View, CA).

In selected experiments, TEC were seeded on six-well plates and Megalin siRNA, or relative control siRNA (80 pM) was introduced according to the manufacturer’s instructions (Santa Cruz Biotechnology). After 48 h, the effective suppression of specific mRNA and proteins was verified by RT-PCR, by WB and by citofluorimetric analysis (unpublished data). Subsequently, the cells were used to evaluate proliferation and apoptosis.
